# Importance of exercise stress testing in evaluation of unexplained cardiac arrest survivor

**DOI:** 10.1007/s12471-023-01789-w

**Published:** 2023-06-22

**Authors:** Auke T. Bergeman, Tomas Robyns, Ahmad S. Amin, Arthur A. M. Wilde, Christian van der Werf

**Affiliations:** 1 Heart Centre, Department of Cardiology, Amsterdam University Medical Centres, location Academic Medical Centre, Amsterdam, The Netherlands; 2 European Reference Network for Rare and Low Prevalence Complex Diseases of the Heart (ERN GUARD-Heart), Brussels, Belgium; 3 Department of Cardiovascular Diseases, University Hospitals Leuven, Leuven, Belgium; 4 Amsterdam Cardiovascular Sciences, Heart Failure and Arrhythmias, Amsterdam, The Netherlands

**Keywords:** CPVT, Exercise stress testing, Sudden cardiac arrest

## Abstract

**Background:**

In sudden cardiac arrest survivors without an immediately identifiable cause, additional extensive yet individualised testing is required.

**Methods:**

We describe 3 survivors of sudden cardiac arrest in whom exercise stress testing was not performed during the initial hospital admission.

**Results:**

All 3 patients were incorrectly diagnosed with long QT syndrome based on temporary sudden cardiac arrest–related heart rate–corrected QT interval prolongation, and exercise stress testing was not performed during the initial work-up. When they were subjected to exercise stress testing during follow-up, a delayed diagnosis of catecholaminergic polymorphic ventricular tachycardia (CPVT) was made. As a result, these patients were initially managed inappropriately, and their family members were initially not screened for CPVT.

**Conclusion:**

In sudden cardiac arrest survivors without an immediately identifiable cause, omission of exercise stress testing or erroneous interpretation of the results can lead to a delayed or missed diagnosis of CPVT, which may have considerable implications for survivors and their family.

## What’s new?

- Despite guideline recommendations, exercise stress testing is not infrequently omitted in survivors of sudden cardiac arrest (SCA).
- Long QT syndrome, rather than catecholaminergic polymorphic ventricular tachycardia, may be erroneously diagnosed based on heart rate–corrected QT interval QTc prolongation following SCA.
- A systematic approach to testing, including exercise stress testing, in SCA survivors allows for disease-specific treatment and family screening.

## Introduction

Survivors of sudden cardiac arrest (SCA) due to ventricular fibrillation (VF) in whom any overt cardiac, toxicological, metabolic and other noncardiac aetiologies have been ruled out are labelled as having idiopathic VF. This is a diagnosis of exclusion and requires a rigorous and comprehensive investigation. However, prior studies have indicated that the work-up of unexplained SCA survivors is often incomplete [1, 2]. Simple investigations such as exercise stress testing may be omitted or interpreted incorrectly. In fact, the results of an exercise stress test may even lead to an alternative diagnosis and, of equal importance, to alternative therapeutic choices.

Herein, we describe 3 patients who presented after an ostensibly unexplained SCA and in whom catecholaminergic polymorphic ventricular tachycardia (CPVT) was ultimately diagnosed through (delayed) exercise stress testing during follow-up, highlighting the complexity and potential oversights in reaching the correct diagnosis.

## Cases

### Case A

A 40-year-old woman with no past medical history was dancing at a party when she suddenly collapsed and became unresponsive. Cardiopulmonary resuscitation was initiated immediately, and after a single shock from a nearby automated external defibrillator, return of spontaneous circulation was achieved. She reported experiencing dizziness immediately before collapsing but denied having had any other complaints and had been in her usual state of health prior to the event. She drank alcohol socially and did not have a history of tobacco or drug use. Notably, she had experienced syncope while cycling when she was 15 years old. Nearly 30 years ago, her younger brother had died suddenly at the age of 10 years while swimming in the pool. Autopsy did not reveal any abnormalities, and the parents and sister had resting 12-lead electrocardiograms (ECGs) recorded, which were normal. They were not referred to a cardiologist for additional screening.

Upon arrival at the emergency department, the patient was alert and oriented. Her vital signs were within normal limits. The 12-lead ECG revealed sinus tachycardia and no abnormalities other than a heart rate–corrected QT interval (QTc) duration of 531 ms. Blood tests showed a mildly reduced potassium level of 3.3 mmol/l (reference values: 3.5–5.0) and elevated levels of aspartate aminotransferase (347 U/l; reference value: < 45) and alanine aminotransferase (311 U/l; reference value: < 50). Other serum electrolytes, haematology tests and creatinine were within normal limits.

She was admitted to the Cardiac Care Unit for monitoring and further evaluation. A repeat ECG, recorded a few days after admission, showed borderline QTc prolongation of 462 ms. Transthoracic echocardiography indicated normal left and right ventricular function and no valvular pathology. Computed tomographic angiography (CTA) showed normal coronary anatomy without evidence of atherosclerotic coronary artery disease. Additionally, cardiac magnetic resonance imaging (CMR) was performed, which revealed mild biventricular dilatation only.

The patient was diagnosed with congenital long QT syndrome (LQTS) and received a dual-chamber implantable cardioverter-defibrillator (ICD). She was treated with metoprolol 100 mg per day for suspected LQTS and ivabradine 7.5 mg twice a day because of sinus tachycardia, after which she was discharged. Subsequently, she was referred to our centre for a second opinion and genetic testing. The first exercise stress test, which was performed while she was being treated with metoprolol, revealed multifocal ventricular ectopy, including polymorphic couplets and triplets, leading to a clinical diagnosis of CPVT. As there was no exercise-induced QTc prolongation and the resting ECG showed a QTc of 443 ms, LQTS was considered less likely. Genetic testing of arrhythmia-associated genes identified 2 compound heterozygous *CASQ2* variants: c.164A>G p.(Tyr55Cys) and c.115G>A p.(Glu39Lys), which seemingly confirmed the diagnosis of CPVT.

Metoprolol and ivabradine were replaced by propranolol 80 mg per day and flecainide 200 mg per day. The nominal ICD settings were modified to a VF-only zone starting at a heart rate of 250 bpm and with the longest possible time to therapy (100 VV intervals < 240 ms by counting in bins). After achievement of complete ventricular ectopy suppression during repeated exercise stress tests and extensive discussion with the patient, it was decided to explant the ICD, which had not intervened during the 9 months it was in place. In the following 7 months, up to the last follow-up visit, no arrhythmic events occurred.

### Case B

An 18-year-old man presented to the emergency department after sustaining a SCA based on VF during an argument. He had a history of mild intellectual disability and autism spectrum disorder, and he had been in his usual state of health, without any prodrome prior to the event. The ECG upon arrival showed sinus tachycardia, diffuse ST-segment depression, which was most pronounced in the precordial leads, and a QTc of 518 ms. Laboratory testing revealed a potassium level of 2.9 mmol/l. He was given routine postarrest care, including targeted temperature management. A transthoracic echocardiogram was normal, whereas isoproterenol testing showed isolated ventricular ectopic beats. Exercise stress testing was not performed. For suspected acquired LQTS secondary to hypokalaemia, spironolactone 12.5 mg per day and bisoprolol 2.5 mg per day were prescribed, and the patient was discharged without insertion of an ICD.

Two years after the index event, the patient experienced 2 episodes of syncope. After the first event, he was found unconscious on the side of the road with urinary incontinence and central cyanosis. He had regained consciousness spontaneously when the emergency medical services arrived but had noticeable bradyphrenia, from which he recovered slowly during the following 24 h. Bisoprolol treatment had been discontinued prior to this event. An epileptic seizure was suspected. The patient was normokalaemic and no cardiac investigations were performed at this time. Five months later, he collapsed again while walking to a bus stop. A witness observed convulsive muscle contractions. In the emergency department, the seizures were controlled with sedatives and a brain computed tomography was performed, which did not show any intracranial pathology. During this admission, an electrophysiological study was performed without a ventricular stimulation protocol, which did not reveal any significant abnormalities. Valproate was initiated for suspected epilepsy, and bisoprolol 5 mg per day was restarted, but the clinical diagnosis remained unclear.

**Fig. 1:**
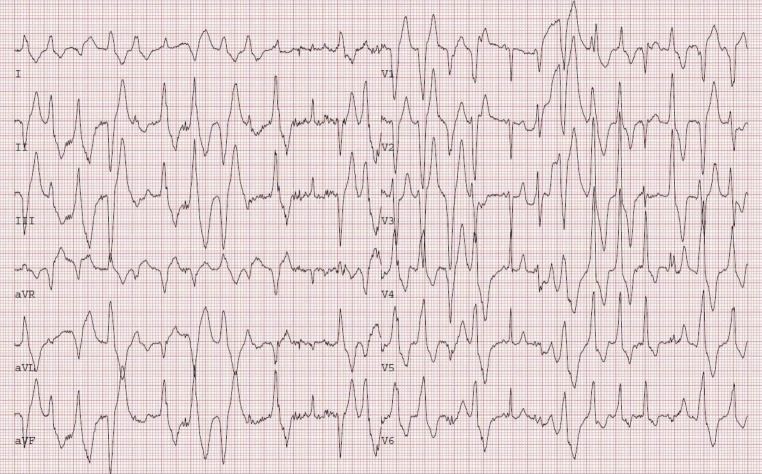
Exercise stress test showing nonsustained polymorphic ventricular tachycardia during peak exercise (case B)

Six years after the sentinel SCA, an exercise stress test was performed—while the patient was being treated with bisoprolol 1.25 mg per day—as part of a routine cardiological examination, which showed nonsustained polymorphic and bidirectional ventricular tachycardia (Fig. 1). CPVT was now suspected, which was later confirmed when genetic testing revealed the pathogenic *RYR2* c.14311G>A p.(Val4771Ile) variant. During the following years, bisoprolol was incrementally increased to 10 mg daily due to polymorphic nonsustained ventricular tachycardia on exercise stress testing, and although this persisted, it was decided not to escalate therapy as the patient had been asymptomatic since the diagnosis. No arrhythmic events occurred during the follow-up of 12 years.

### Case C

A 21-year-old man suddenly collapsed while running. Emergency medical services were alarmed by his mother, who witnessed the event. SCA based on VF was identified, from which he was successfully resuscitated, and he was subsequently transferred to a nearby hospital. The patient had no past medical history, was not taking any medication and did not have a history of tobacco, alcohol or drug use. He later reported he did not have any prodromal symptoms. The family history was remarkable: his brother had died suddenly while on an assault course at the age of 19 years, and his great-uncle had died suddenly while wrestling when he was 32 years old. The admission ECG showed sinus bradycardia at 50 bpm and was notable for a QTc of 529 ms. Laboratory tests were unremarkable; particularly, electrolyte levels were all within normal limits.

As the patient was comatose, he was intubated and admitted to the Intensive Care Unit for targeted temperature management. A transthoracic echocardiogram was performed, showing normal left ventricular function and mild mitral regurgitation. CTA revealed normal coronaries, and CMR showed no abnormalities other than a borderline elevated right ventricular end-diastolic volume index of 117 ml/m^2^ (reference values: 61–121 [3]). The patient was diagnosed with congenital LQTS, and metoprolol 25 mg once daily was started. He recovered well, and his hospital stay was without complications. It was decided to insert a dual-chamber ICD, after which he was discharged.

**Fig. 2:**
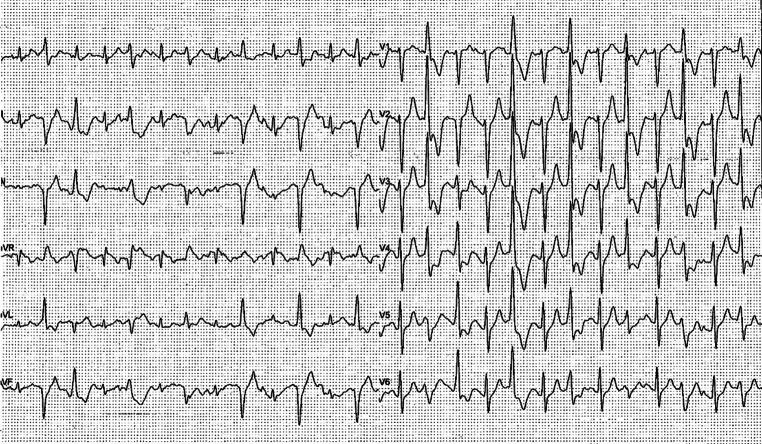
Exercise test showing bigeminal ventricular ectopic beats (case C)

During follow-up, he was referred to an expert centre and underwent an exercise stress test, which showed ventricular ectopic beats in bigeminy as well as couplets (Fig. 2). Genetic analysis of the *RYR2 *gene revealed the *RYR2-*c.848+1G>A variant of unknown significance. Because of a high degree of suspicion of CPVT, flecainide 150 mg daily was initiated in addition to the metoprolol. A series of subsequent exercise tests on this drug regimen demonstrated complete suppression of the ventricular ectopy. This result, combined with multiple ICD-related complications including inappropriate shocks and lead fracture, prompted explantation of the ICD. Three years later, successful cardiac sympathetic denervation was performed after ventricular ectopic beats in bigeminy and couplets were observed on the exercise stress test despite maximally tolerated doses of metoprolol 100 mg per day and flecainide 200 mg per day. During follow-up, he experienced one episode of syncope, which was classified as reflex syncope rather than arrhythmic syncope. There have been no arrhythmic events during the subsequent 5‑year follow-up.

## Discussion

We have described 3 patients who suffered a SCA due to VF for which no obvious initial cause was identified after the initial work-up, illustrating that correctly identifying the aetiology of SCA can be challenging. In these cases, congenital or acquired LQTS was initially diagnosed based on QTc prolongation observed on ECGs immediately after the event, and ICDs were inserted in 2 cases. During the initial hospital admission, exercise stress testing was not performed, even though the events occurred during either exertion or emotion in all 3 patients. Ultimately, a diagnosis of CPVT was established after performing exercise stress testing during follow-up.

CPVT is a rare genetic arrhythmia syndrome that potentially causes malignant bidirectional or polymorphic ventricular arrhythmias. It is diagnosed in the presence of either a (putative) pathogenic mutation in a CPVT-associated gene or catecholamine-induced polymorphic ventricular arrhythmia in the absence of resting ECG abnormalities, structural heart disease and significant coronary artery disease [4]. Ventricular arrhythmias in CPVT are typically provoked by exercise or emotion. Patients may present with palpitations, syncope or SCA, all triggered by adrenergic stimuli.

### Diagnosing CPVT

Several pitfalls can make diagnosing CPVT difficult. For instance, CPVT shares some characteristics with congenital LQTS type I, particularly because swimming and diving are recognised triggers for arrhythmic events in both conditions [5]. However, case A supports previous observations that not all arrhythmic events occur in this context [6]. CPVT should therefore be considered even in patients who experience arrhythmic events in the absence of a typical stress-inducing context, particularly in young individuals. In addition, interpretation of QTc prolongation following SCA is complex. The QTc is often prolonged directly after SCA due to several factors, and QTc prolongation should not be used to diagnose LQTS at this stage [7]. Normalisation of the QTc may take several days.

**Fig. 3:**
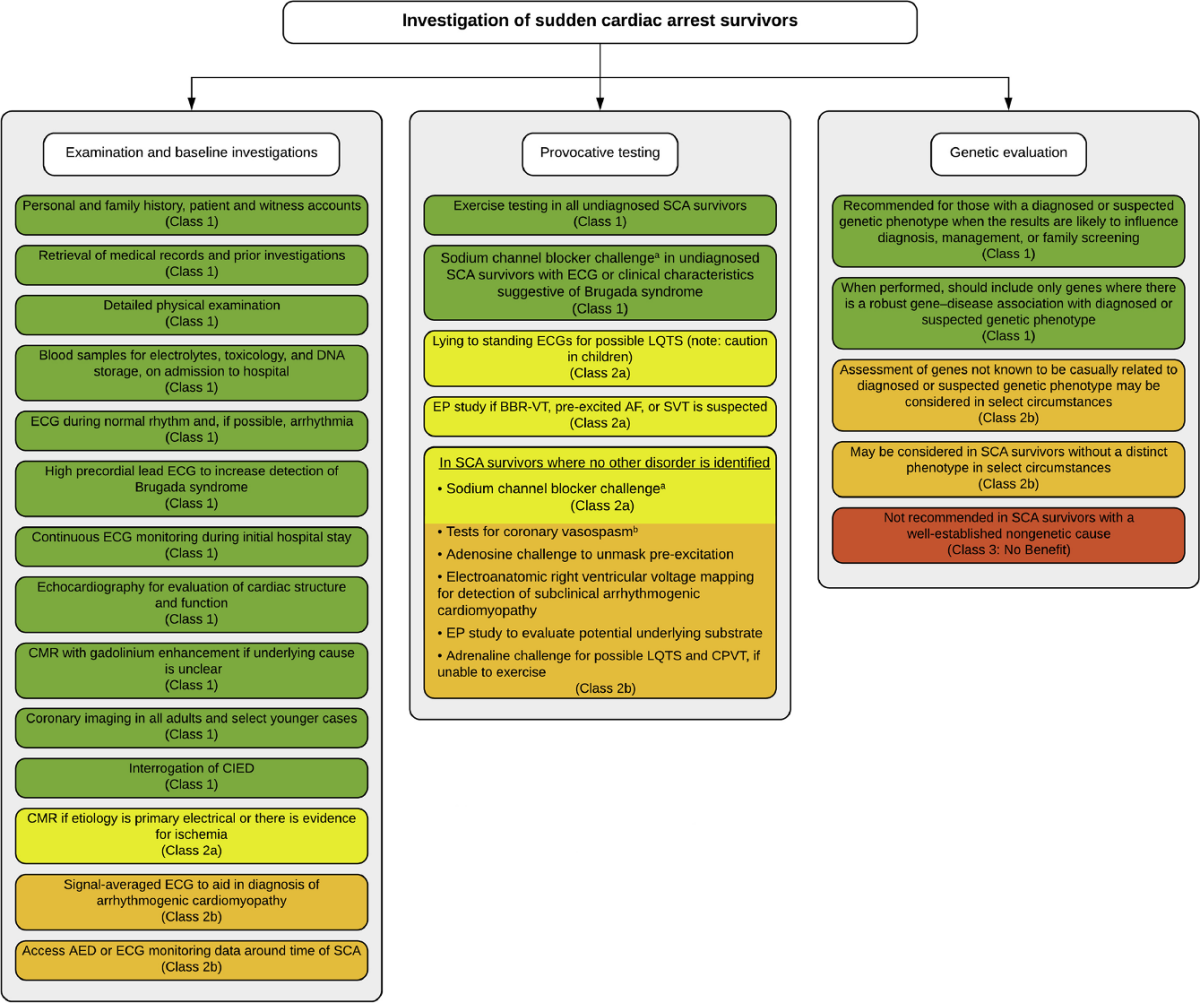
Recommended investigations in sudden cardiac arrest survivors. *AED* automated external defibrillator, *AF* atrial fibrillation, *BBR-VT* bundle branch reentry ventricular tachycardia, *CIED* cardiovascular implantable electronic device, *CMR* cardiac magnetic resonance imaging, *CPVT* catecholaminergic polymorphic ventricular tachycardia, *ECG* electrocardiogram, *EP* electrophysiological, *LQTS* long QT syndrome, *SCA* sudden cardiac arrest, *SVT* supraventricular tachycardia. Colours reflect a class I (strong) recommendation (*green*), class IIa (moderate) recommendation (*yellow*), class IIb (weak) recommendation (*orange*) and no benefit (*red*). This figure was reprinted under a CC-BY licence from: Stiles MK, Wilde AAM, Abrams DJ, et al. 2020 APHRS/HRS expert consensus statement on the investigation of decedents with sudden unexplained death and patients with sudden cardiac arrest, and of their families. Heart Rhythm. 2021;18:e1–50

A recent consensus report provides guidance to clinicians in the assessment of survivors of SCA [8]. Baseline testing should include a 12-lead ECG, echocardiography, coronary imaging and, in unexplained cases, CMR. If the aetiology is not identified, provocation tests should be considered, including exercise stress testing, supine-to-standing ECG, sodium channel blocker challenge and acetylcholine provocation (Fig. 3). As shown in the cases we have described, especially exercise stress testing is not infrequently omitted [1, 2], despite its simplicity and the wealth of information it provides. Pharmacological provocation using epinephrine or isoprenaline may be an alternative test but only in patients who are unable to exercise. Exercise stress testing enables not only recognition of complex ventricular ectopy as may be seen in CPVT, but QTc prolongation during the recovery phase in LQTS may also be identified [9]. Case B exemplifies that exercise stress testing may reveal more diagnostic information than drug testing with either isoprenaline or epinephrine [10].

Diagnostic challenges notwithstanding, it is pivotal to recognise CPVT in a patient presenting with syncope or SCA. A correct diagnosis allows for disease-specific patient management and facilitates family screening, the importance of which cannot be understated in CPVT, as relatives are also at risk of sudden cardiac death [5, 11].

### Treatment

Therapeutic interventions in CPVT are aimed at preventing arrhythmic events while minimising side effects. Beta-blockers, particularly nadolol and propranolol [12], remain the cornerstone of medical therapy in CPVT. Flecainide can be added in patients with insufficient control of ventricular arrhythmias or beta-blocker intolerance. When ventricular arrhythmias persist despite optimal and maximally tolerated medical therapy, left cardiac sympathetic denervation should be considered [13].

Although ICD implantation is generally indicated in survivors of SCA without a reversible cause, consideration is required, as not all CPVT patients may benefit from an ICD. In this population, there are certain disadvantages to ICD therapy. Firstly, ICD shocks, both appropriate and inappropriate, may provoke an electrical storm due to pain and distress and the resultant catecholamine surge, which may occasionally even be fatal. In addition, antitachycardia pacing and ICD shocks are often ineffective in treating polymorphic and bidirectional VTs, as these are not arrhythmias based on reentry [14, 15]. Lastly, device-related complications, such as infection, lead malfunction and inappropriate shocks, can occur in all patients with an ICD but seem to be more prevalent in young (CPVT) patients, which further contributes to an unfavourable safety profile.

One observational study showed that ICD implantation in previously undiagnosed and untreated CPVT patients presenting with a sentinel SCA—similar to the 3 cases presented—was not associated with a reduction in sudden cardiac death [16]. These data were the main reason to perform ICD explantation in cases A and C, following extensive discussions with the patients or their legal representatives.

## Conclusion

A high degree of suspicion of CPVT in survivors of SCA without an immediately identifiable cause is crucial, especially in the young. The cases presented underscore the importance of systematic and standardised testing, including exercise stress testing, which has substantial yield and enables disease-specific therapy and management decisions, which in turn can be life-saving for patients and their relatives.
